# Editorial: The role of the microbiome in head and neck cancer

**DOI:** 10.3389/fonc.2025.1545067

**Published:** 2025-01-17

**Authors:** Fabio Cumbo, Jayadev Joshi, Dietmar Thurnher, Anastasios Maniakas

**Affiliations:** ^1^ Center for Computational Life Sciences, Lerner Research Institute, Cleveland Clinic, Cleveland, OH, United States; ^2^ Department of Otolaryngology, Head and Neck Surgery, Medical University of Graz, Graz, Austria; ^3^ Department of Head and Neck Surgery, The University of Texas MD Anderson Cancer Center, Houston, TX, United States

**Keywords:** microbiome, head and neck cancer, HPV, oropharynx, editorial

Head and neck cancers (HNCs) are among the most challenging malignancies worldwide, often associated with significant morbidity and mortality (1). In recent years, the role of the microbiome in these cancers has emerged as a promising area of research, offering insights into previously unexplored mechanisms of cancer initiation, progression, and response to treatment. Growing evidence suggests that alterations in the oral and gut microbiome can influence cancer biology through various mechanisms, including the induction of chronic inflammation, modulation of the immune system, and the production of carcinogenic metabolites. These processes create a permissive environment for tumor growth while potentially impairing the immune system’s capacity to detect and eliminate cancer cells. Furthermore, the role of intratumoral microbiome remains unclear in HNC, and research suggests it may play a pivotal role in immune evasion.

Despite the growing body of evidence supporting the microbiome’s involvement in HNCs, significant gaps remain in our understanding of its functional roles and clinical relevance. For instance, identifying specific microbial biomarkers linked to these cancers and unraveling their mechanistic contributions are critical steps toward harnessing the microbiome for diagnostic and therapeutic purposes. Furthermore, understanding the interplay between microbial communities and cancer therapies could shed light on their influence on treatment efficacy, resistance, and patient outcomes. Lastly, the interaction between the microbiome and the intrinsic tumor metabolic activity remains to be clarified.

This Research Topic has aimed to entice the scientific community to begin filling these knowledge gaps by compiling studies that explore the intricate relationship between the microbiome and HNCs. These contributions have focused on identifying specific microbial biomarkers, elucidating their roles in disease mechanisms, and evaluating their potential in diagnostics and therapy. Additionally, studies investigating the interaction between the microbiome, immune system, and cancer treatments have offered new insights into how microbial communities may shape therapeutic outcomes.

In this Editorial, we highlight five articles that exemplify the diverse and innovative research contributions to this Research Topic.

The study by Lian et al. investigates the role of the gut microbiome in HNC, using genome-wide association studies and Mendelian randomization. They identified specific microbial compositions that have causal effects on HNC: The family *Peptococcaceae.id.2024* is linked to a reduced risk, while the genera *DefluviitaleaceaeUCG-011.id.11287*, *Gordonibacter*, and *Methanobrevibacter* are associated with an increased risk. These findings indicate potential biomarkers and therapeutic targets, offering potential new directions for diagnosis, prevention, and treatment of HNC.

The study by Sun et al. employed a two-sample bidirectional Mendelian randomization approach to explore the causal relationship between gut microbiota and oral cavity cancer (OCC). Leveraging genome-wide association study data from over 18,000 participants for gut microbiota and 372,000 individuals for OCC, the researchers identified several bacterial taxa with significant causal effects on OCC risk. The findings revealed that taxa such as *Burkholderiales* (order), *Alcaligenaceae* (family), and *Desulfovibrio* (genus) are associated with an increased risk of OCC, whereas others, including *Bacteroidales* (order), *Clostridium sensu stricto 1* (genus), and *Eggerthella* (genus), demonstrated protective effects. The study also highlighted the complexity of these relationships, with some taxa influencing OCC through multiple biological pathways. These results emphasize the dual role of gut microbiota in both promoting and preventing OCC, also suggesting the potential of specific microbial taxa as biomarkers for risk stratification or as targets for therapeutic intervention. The authors recommend further mechanistic studies to unravel the biological pathways underlying these associations and to refine microbiome-based strategies for OCC prevention and treatment.

Chung et al. proceeded to shed light on the complex interactions between HPV and key microorganisms in the oropharynx, particularly *Neisseria gonorrhoeae* and *Chlamydia trachomatis*. Specifically, they elaborate on the potential crosstalk mechanism, identifying interactions that may contribute to persistent HPV infection in the oropharynx, and subsequent carcinogenesis. The key mechanisms they identify are mucosal barrier disruption, induction of DNA damage, and immune response modulation. These mechanisms may create an environment conducive to HPV persistence and squamous epithelium oncogenic transformation. This manuscript, led by Dr. Albert Han and his lab, helps direct future research efforts towards elucidating specific mechanisms of HPV-microbial crosstalk and exploring potential therapeutic interventions targeting the microbiome.

Liu et al. reported their findings on two biomarkers, p16 status (positive or negative) and Ki67 expression levels (0-35%, 36-70%, 71-100%), and their prognostic significance in laryngeal carcinoma from a single center. Per their findings, high levels of Ki67 expression was a clear predictor of worse overall survival, with a 2-fold death rate compared to patients harboring tumors with low Ki67 expression, while p16 status was not associated with disease or overall survival outcomes. Although HPV infection of the larynx has been associated with carcinogenesis, the relationship is not as well-established as it is with the oropharynx. As a frequently-used surrogate to HPV, p16 remains an important biomarker that should undergo further testing in the setting of laryngeal cancer.

In the study by Zhou E. et al., authors utilized bidirectional two-sample Mendelian randomization analyses with GWAS and FinnGen databases to examine 731 immune cell features and their association with HNMN risk. Immune escape and immunosuppression are pivotal in the development and progression of head and neck malignant neoplasms (HNMN). However, prior studies on the link between immune cells and HNMN have been inconclusive. In their analysis, after correcting for the false discovery rate, three immune cell phenotypes showed significant correlations with HNMN risk: CD28-CD8+ absolute cells, CD3+ secreting Tregs, and CD3+ resting Tregs. Their findings, supported by sensitivity analyses, highlight potential causal relationships between specific immune cell phenotypes and HNMN vulnerability. Overall, these findings offer insights into the tumor microenvironment and aid in the development of immunotherapies targeting checkpoint inhibitors.

In conclusion, these studies are a clear example of how complex HNC development and outcomes remain, and demonstrate the potential multifaceted role of the microbiome and other tumor microenvironment factors may play. These studies set the stage for future investigations that may lead to innovative diagnostic and therapeutic strategies.
